# YSTAFDB, a unified database of material stocks and flows for sustainability science

**DOI:** 10.1038/s41597-019-0085-7

**Published:** 2019-06-07

**Authors:** Rupert J. Myers, Barbara K. Reck, T. E. Graedel

**Affiliations:** 1 Present Address: School of Engineering, The University of Edinburgh, King’s Buildings, Sanderson Building, Edinburgh, EH9 3FB United Kingdom; 2 School of Forestry & Environmental Studies, Yale University, 195 Prospect St, New Haven, 06511 Connecticut United States

**Keywords:** Environmental impact, Engineering, Industry, Technology, Sustainability

## Abstract

We present the Yale Stocks and Flows Database (YSTAFDB), which comprises most of the material stocks and flows (STAF) data generated at the Center for Industrial Ecology at Yale University since the early 2000s. These data describe material cycles, criticality, and recycling in terms of 62 elements and various engineering materials, e.g., steel, on spatial scales and timeframes ranging from cities to global and the 1800s to ca. 2013. YSTAFDB integrates this diverse collection of STAF data, previously scattered across various non-uniformly formatted electronic files, into a single data structure and file format. Here, we discuss this data structure as well as the usage and formatting of data records in YSTAFDB. YSTAFDB contains 100,000+ data records that are all situated in their systems contexts, with additional metadata included as available. YSTAFDB offers a comprehensive basis upon which STAF data can be accumulated, integrated, and exchanged, and thereby improves their accessibility. Therefore, YSTAFDB facilitates deeper understanding of sustainable materials use and management, which are key goals of contemporary sustainability science.

## Abstract (summary)

**Table:** 

Design Type(s)	data integration objective • source-based data analysis objective
Measurement Type(s)	reference material
Technology Type(s)	digital curation
Factor Type(s)	Material • temporal_interval • geographic location
Sample Characteristic(s)	Earth (Planet) • anthropogenic habitat

Machine-accessible metadata file describing the reported data (ISA-Tab format)

## Background & Summary

Sustainability science studies are becoming increasingly data intense. Concurrently, the need for these studies is growing amid heightened concerns for issues such as material scarcity, climate change, waste reduction, and equitable economic growth and development. A sustainability science study relies upon material stocks and flows (STAF) data to describe how materials and related properties such as mass, energy, and money are used in its system of interest. Such systems may be anthropogenic or natural, describe a supply chain of a company, a food web comprising endangered marine species, or environmental emissions of one or more energy generation technologies. STAF data may describe full or partial (life) cycle(s) of one or more reference material(s) in these systems, e.g., iron (Fe) in a study involving static material flow analysis (MFA)^1^; transport-related goods and elements in a study involving dynamic MFA^2^; and battery products and components in a study involving life cycle assessment (LCA)^3^. Analyses of material efficiency^4^, criticality (i.e., the risk of material unavailability)^5^, and recycling^6^ may analyze these data directly to describe material systems; alternatively, STAF data may be applied to characterize impacts and assess environmental damage (or benefit) of product systems^7,8^.

Sustainability science studies are constrained by the limited availability of STAF data and their ease of (re)use. The availability of useable STAF data is compounded by multiple factors, including: (1) the high and increasing interconnectedness and complexity of anthropogenic and natural systems; (2) the relatively recent development and usage of computational approaches to sustainability science^9^; and (3) the ongoing establishment of infrastructure to support these approaches. Current efforts into developing research practices that enable higher data accessibility, transparency, and efficient re-use of data^10,11^ may eventually alleviate some of the current challenges. Presently, STAF data are obtained from diverse (public and confidential) sources, structured in different formats, described with different terminology, and produced using different methodologies. Consequently, results from apparently similar sustainability science studies may vary significantly. These issues make it challenging to unify and build upon STAF data, and also to verify the reliability of the studies that use them. Therefore, a comprehensive and openly accessible STAF database would be a highly desirable resource for sustainability science. It would facilitate re-use of STAF data and lead to more reliable and higher quality sustainability science analyses and assessments.

This paper presents the Yale STAF Database (YSTAFDB), which contains most of the STAF data associated with studies of material cycles, recycling, and criticality conducted by Graedel and colleagues at the Center for Industrial Ecology at Yale University since the early 2000s. These 100,000+ data records were previously reported in various formats across 60 publications (e.g.^5,12–15^). YSTAFDB is unique in its diversity of STAF data, which cover ~75% of the periodic table of elements excluding those that are non-primordial and radioactive (e.g., polonium [Po]), various engineering materials, spatial scales ranging from local to global, and timescales from the early 1800s to ca. 2013. The data are recorded in a consistent manner within a material cycle ‘systems context’^16^. Therefore, YSTAFDB presents a step toward overcoming the limited accessibility that has resulted from the incomplete availability of the STAF data, by integrating them into a single data structure and database format.

It is useful to indicate the broader context of the material cycles, recycling, and criticality studies from which data records in YSTAFDB originate. These data result from an approximately two decades long ‘Stocks and Flows (STAF) Project’ that sought to quantitatively describe anthropogenic material cycles. However, the STAF Project was conducted among a wider industrial ecology research community effort to understand material systems: this community notably applied MFA as a basis for analysis of environmental and policy issues. Other exemplars of this community effort include: work done by Baccini, Brunner, and colleagues, who were key players in defining MFA methodology in a systematic way and applying it to understand the metabolism of anthropogenic systems such as cities and local regions^17,18^; and also coordinated studies conducted at institute (e.g., Wuppertal Institute) to international (e.g., Organisation for Economic Cooperation and Development) levels to improve material efficiency, particularly at the national economy scale. For example, the Japanese Government initiative to develop a ‘sound material-cycle society’^19^ developed and applied MFA indicators to measure and drive its resource productivity agenda^20,21^. This history indicates that the STAF Project and YSTAFDB comprise one key part of a landscape of MFA studies and STAF data.

The comprehensive and consistent nature of data records in YSTAFDB facilitates its use as a key STAF data resource. For example, YSTAFDB may be used to accumulate, structure, and enhance STAF data in the future to facilitate sustainability analyses and assessments, and thus to help identify and approach sustainable development. These additional STAF data may be sourced from historical work such as those described above, as well as (more) contemporary and future studies. We are working toward this goal through this initial release of YSTAFDB, which we provide and discuss as a set of comma separated value (csv) files. The following sections of this paper describe the methods used to create YSTAFDB, its properties and the data records in the csv files as released, and its usage.

## Methods

YSTAFDB contains data from 60 publications that are broadly grouped into three categories: (1) material cycles; (2) criticality; and (3) recycling. Brief descriptions of the methods used to produce these data are provided here; complete descriptions of these methods are available elsewhere^16,22^. Our approach is to record the material cycles data within their systems contexts, utilizing the Unified Materials Information System (UMIS)^23^ as a data structure, also described here.

### Material cycles

**Fig. 1:**
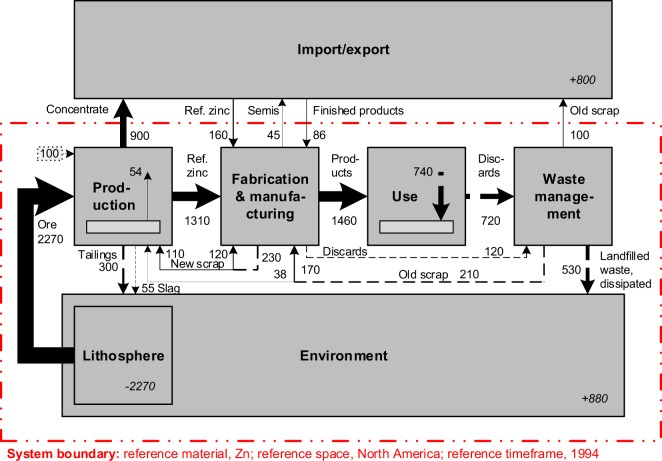
An exemplary quantified material cycle in block flow diagram format, the North American zinc (Zn) cycle circa 1994. Adapted from^41^. The system boundary (reference material, zinc; reference space, North America; reference timeframe, 1994) is represented by the red dashed double dotted line, flows by arrows, and processes by boxes. The estimated confidence level in the flow quantities for dashed arrows is lower than for solid arrows. The small dotted box (containing the text ‘100’) and arrow towards the top left of the figure represents the quantitative discrepancy between the available STAF data and the application of mass conservation to those data. Quantities are shown in Gg Zn (per year).

The first step to produce a material cycle (Fig. 1) is to define its goal and scope. This may be, e.g., to quantify how much and in what form Cu is used across all major anthropogenic activities in 2018. A system of interest, i.e., a system that corresponds to the goal and scope, is defined by a system boundary that comprises a reference material, a reference timeframe, and a reference space. For the example described here, the system boundary may be represented by Cu (reference material), the year 2018 (reference timeframe), and geographic entity (e.g., North America). Reference materials may be elements (e.g., copper [Cu]), engineering materials (e.g., brass), specific products (e.g., a Ford Focus), product groups (e.g., cars), etc.

The system is then populated with processes, which are linked together by flows in sufficient detail to satisfy the project goal and scope. Processes involve one or more of the following properties: (1) transformation, to transform material from one type to another; (2) distribution, to distribute material from one location to another; (3) and/or storage, to withdraw and/or deposit material from or into a stock. Distributive processes may be used as modelling constructs or conceptual tools in material cycles to simplify the underlying calculations and data visualisations. They are sometimes depicted as ‘market processes’ (e.g.^1,24^). Processes and flows are often specified to cumulatively represent a (life) cycle of a reference material of interest, e.g., the global socioeconomic metabolism with a reference material of ‘all materials’^25^. In the Cu example used in the previous paragraph, processes and flows would be specified to describe the anthropogenic Cu cycle, including the production of engineering materials (e.g., Cu metal), fabrication and manufacturing (e.g., of Cu wire), use (e.g., in buildings), waste management and recycling (e.g., of old wire scrap), and the relationships that these processes have with the environment (e.g., mining of chalcopyrite ore) and Cu stocks (e.g., unmined chalcopyrite ore). Quantitative data are then collected from various sources to describe as many processes, stocks, and flows as possible. The processes, stocks, and flows without quantitative data are termed ‘data gaps’.

Data gaps can be reduced or filled by applying mass and energy conservation, assumptions, or estimations to the reference material cycle. This is typically done in one of two ways:

1. By re-assessing and re-specifying the processes and flows initially used to define and populate the system of interest, and obtaining additional quantitative data for these re-specified processes and flows^16^. This procedure may be iterated many times before the data gaps are sufficiently reduced and the system is described to the level of detail desired by the analyst, i.e., until it fulfills the project goal and scope. Alternatively, the project goal and scope may be redefined to accommodate the prevailing availability of STAF data. This method leads to material cycles specified in terms of user-defined processes and flows, and thus often requires reported data to be reinterpreted by the analyst.
2. By estimating data gaps, including the uncertainties of these estimates, without re-specification of processes and flows^26^. This method may lead to material cycle models with relatively poorer initial accuracy, although ‘incremental’ refinement of data used to fill data gaps through use of additional data sources would eventually lead to more reliable, accurate, and transparent models than those produced using the former method. The recently developed incremental method^26,27^ facilitates closer comparison among material cycle models and to data reported by data providers, e.g.^28^.

YSTAFDB contains STAF data for material cycles that were produced using the first method only. However, STAF data generated using both methods can be stored in YSTAFDB – they are distinguished here only to clarify how material cycles may be produced.

### Unified Materials Information System (UMIS)

Material cycles data were structured using the Unified Materials Information System (UMIS)^23^ in YSTAFDB. UMIS is a data structure that can be used to integrate STAF data from various sources into a consistently formatted, flexible, and generalizable system context without loss of information. UMIS does this by labeling STAF data with their positions in their respective systems. These labels uniquely index subsystems, their constituent processes and flows, and also stocks and metadata associated with these processes and flows. In a tree-type hierarchy of processes arranged by specificity, we term the parent a subsystem and its child a process. In doing so, we adopt common informatics terminology (‘parent’, ‘child’, ‘tree’, ‘tables’, ‘mapping tables’, etc.) that is relevant to describing the same types of data systems (e.g., databases) in sustainability science.

**Fig. 2:**
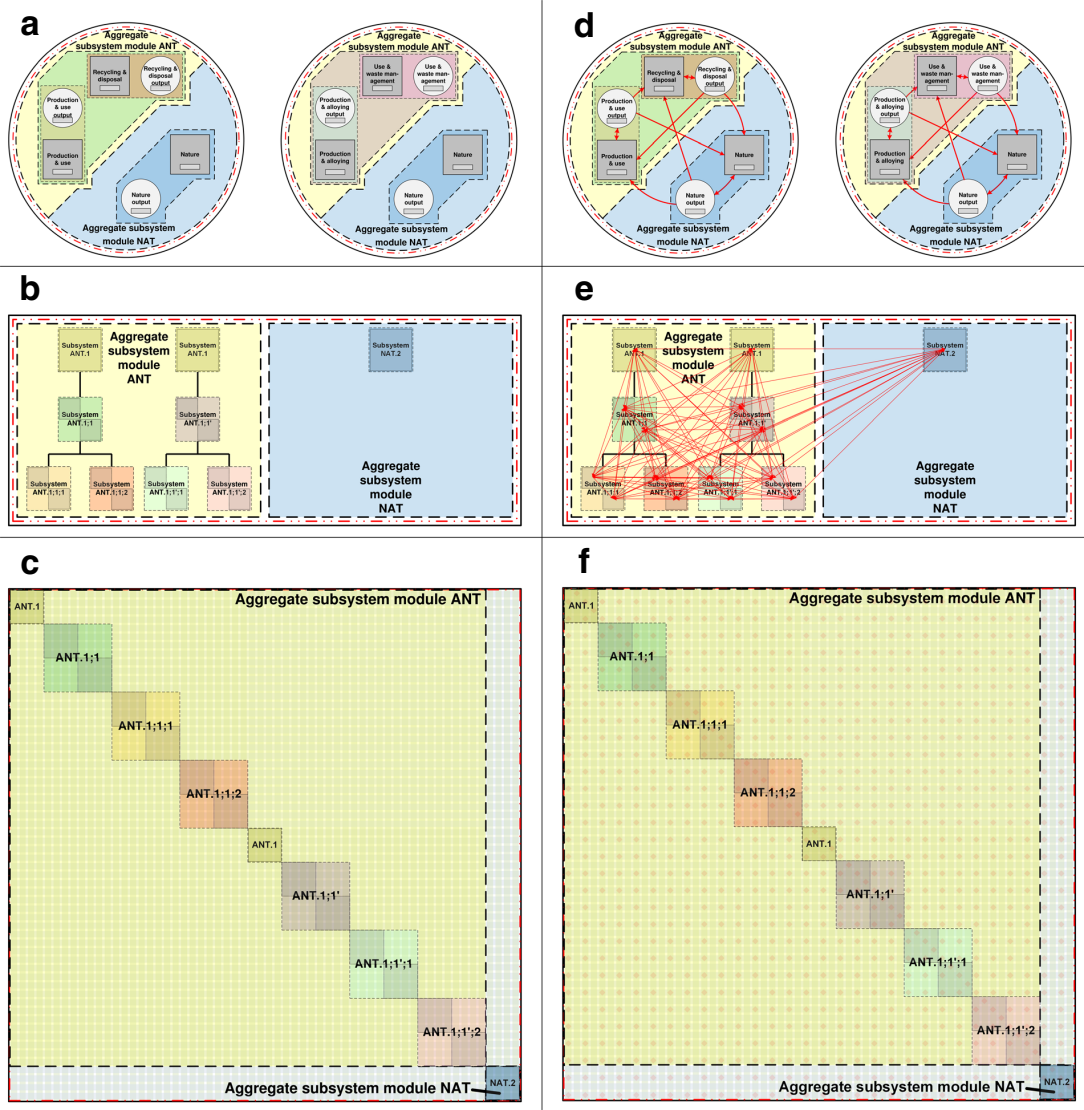
Equivalent representations of UMIS structured STAF data in generic material cycles. Material cycles are shown without flows (**a**–**c**) and with flows. (**d**–**f**) Material cycles are represented in block flow diagrams (**a**,**d**) tree-type UMIS diagrams (**b**,**e**) and matrix-type UMIS diagrams. (**c**,**f**) Subsystems are represented by dashed color-shaded boxes, flows by red diamonds and arrows (in **d**–**f**), processes by grey circles, squares, and rectangles (in **a** and **d**) or grey shaded boxes (in **b**,**c** and **e**,**f**), system boundaries by red dashed double dotted lines, and whole system boundaries by thick black solid lines. Parent-child relationships (among subsystems) are shown as solid black lines in (**b**,**e**) and omitted in (**a**,**c**,**d**,**f**).

Process labels take the form *a*.*b*.*c*.*d*.*e*, where *a* is the reference material, *b* is the aggregate subsystem module abbreviation (representing the material (life) cycle stage), *c* is the subsystem code, *d* is the type of process (transformative (*T*) or distributive (*D*)), *e* is a process code that is unique to each process in each subsystem for reference material *a*, and dots (.) demarcate these five components of process labels. An example process label is *58*.*USE*.*3*.*T*.*1;1*. Flow labels take the form *origin_destination*, where *origin* and *destination* refer to initial and terminal processes for the flow. An example flow label is *1*.*ENV*.*5;1*.*D*.*12;12_1*.*PEM*.*1*.*T*.*1;1*. These UMIS structured data, within their material cycle and systems context, may be visualized completely in UMIS ‘elicitation’ diagrams (Fig. 2).

Figure 2a,d each present two generic material cycles in conventional block flow diagram format (four in total), two with flows omitted (a), and the same two diagrams but with flows included (d). Two generic material cycles are presented in each figure panel (a) and (d) to illustrate how ‘divergent’ disaggregation is conceptually treated in UMIS^23^. The reader can observe that the anthropogenic processes represented in the block flow diagrams within a single figure panel, (a) or (d), are disaggregated differently: in one, a generic ‘anthropogenic’ process is disaggregated into ‘production & use’ and ‘recycling & disposal’ processes; in the other, a generic ‘anthropogenic’ process is disaggregated into ‘production & alloying’ and ‘use & waste management’ processes. We illustrate how UMIS reconciles this case of divergent disaggregation in Fig. 2b,c,e,f, where we equivalently represent the data in these two block flow diagrams into one tree-type (b-c) UMIS diagram and one matrix-type UMIS diagram (e-f). We provide these alternative diagrams to conceptually show how data can be comprehensively and consistently structured in UMIS at the whole system level, in a generic yet unified manner. They are complementary to traditional STAF data visualizations such as block flow and Sankey diagrams.

We purposely distinguish processes and flows data, stocks data, and metadata in order to conceptualize them as three distinct layers in UMIS that together completely describe a material system (e.g., a material cycle). These layers are termed the ‘processes and flows layer’, the ‘virtual reservoir’, and the ‘metadata layer’ in UMIS (see Fig. 4b in^23^; Fig. 2 here shows the ‘processes and flows layer’ only). We distinguish the process and flows layer, which has a direct physical meaning analogous to an input-output table, from the latter two layers that have indirect physical meanings such as uncertainty determinations^23^. This distinction is useful to enhance the comparability of the processes and flows layer in UMIS to the flow-based input-output tables and process matrices that are used in input-output analysis and life cycle assessment.

**Fig. 3:**
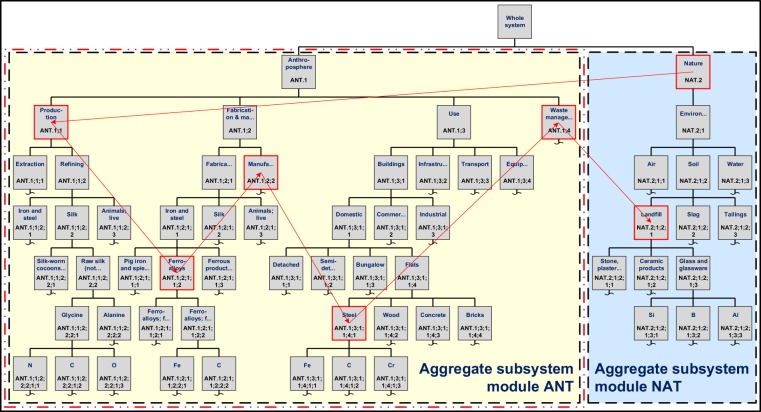
Representative material cycle in a tree-type UMIS diagram for labeled and classified STAF data in YSTAFDB. Example nodes (subsystems) are used to populate the diagram; data for the subsystems shown are not necessarily present in YSTAFDB. Subsystems are shown as grey squares and aggregate subsystem modules as dashed color-shaded boxes. Subsystem labels are in black text, and parent-child relationships (among subsystems) are shown as solid black lines. Subsystem descriptions are in blue text. An example material cycle in this system is depicted as flows (red arrows) among subsystems (red outlined grey squares). All other flows, and all processes, are omitted in this figure. Curly horizontal lines indicate the omission of more disaggregated subsystems in the figure. The system boundary is the red dashed double dotted line. The whole system boundary is the thick black solid line.

Tree-type UMIS diagrams are useful for visualizing STAF data within databases such as YSTAFDB. These diagrams depict ‘trees’ (Figs 2b,e and 3) that may cumulatively represent the entire material cycle (and thus potentially also the whole system). In a previous contribution^23^ we termed these trees ‘material trees’; however, we henceforth refer to these trees as ‘process trees’ because this term is better aligned with their purpose, which is to represent tree-type process hierarchies such as those shown in Fig. 3. Branches in these process trees represent non-overlapping parts of a material cycle, which are often represented in terms of (life cycle) stages (e.g., fabrication and manufacturing). Infinite disaggregation of data is possible in UMIS by specifying child, grandchild, etc., nodes (processes and/or subsystems) in each process tree. Disaggregation is termed ‘consistent’ if each disaggregated node is more specific than its parent node. However, nodes can be disaggregated by specificity in more than one way: e.g., a cars process may be disaggregated into red cars and blue cars processes, or alternatively, that same cars process may be disaggregated into big cars and small cars processes. Here, red/blue and big/small cars occur on the same disaggregation level in the process tree and are not additive. This type of disaggregation is termed ‘divergent’. UMIS uniquely labels consistent and divergent disaggregation such that double counting of data can be avoided in computational modeling of UMIS structured STAF data. Figure 2b,e show tree-type UMIS diagrams for a material system containing divergent disaggregation of data in subsystem ANT.1 (in aggregate subsystem module ANT). Figure 3 shows consistent disaggregation.

The tree-type UMIS diagram in Fig. 3 is simplified by showing labeled subsystems, several flows, and omitting processes. However, all processes and flows can be shown explicitly in tree-type UMIS elicitation diagrams (Fig. 2c,f). Tree-type UMIS elicitation diagrams that include all labeled processes are thus able to concisely describe and visualize classified and labeled STAF data. Therefore, they may be used to comprehensively query data records in STAF databases such as YSTAFDB (processes, flows, stocks, metadata).

UMIS labels of processes and flows in YSTAFDB data records can be parsed to identify and update their locations in material cycles. Parsing of UMIS labels in data records would be needed to accommodate changes to the STAF data structure, e.g., to update them as more data are added to YSTAFDB and/or as subsystem and/or process disaggregation changes. Therefore, we envisage that integrating additional data into YSTAFDB, and use of existing data in YSTAFDB, will be facilitated through the development and application of an internationally standardized classification system for processes and flows (i.e., materials, products, energy, etc. that are distributed among processes). The Harmonized System (HS)^29^ is one such classification system that may be used for this purpose. The North American Industry Classification System (NAICS) is another. The use of a classification system will thus also reduce reinterpretation of reported data.

### Criticality

A criticality assessment characterizes the risk of material unavailability in a system of interest. Criticality assessments are produced using STAF data for material cycles and supplemented by additional data as established by the methodology used (e.g., political stability indicator values^5^).

**Fig. 4:**
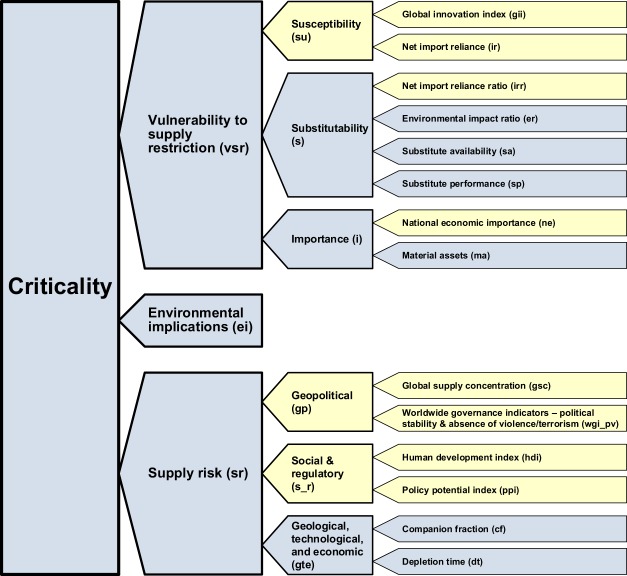
Criticality methodology developed by Graedel and coauthors. Adapted from^5^. Indicators in blue are used in long-term and medium-term assessments; indicators in yellow are used in medium-term assessments only. Corporate level criticality assessment indicators are omitted here. Indicator labels are sized for reader convenience only – weighting is not implied.

Criticality data in YSTAFDB were produced using the methodology developed by Graedel and colleagues^5,22^ (Fig. 4). This methodology defines criticality along three dimensions: (1) supply risk (sr); (2) environmental implications (ei); and (3) vulnerability to supply restriction (vsr). The overall criticality indicator is a linear combination of scores along these three dimensions.

Supply risk characterizes the chance that material supply, from both virgin and secondary resources, may not meet demand. It is characterized as medium-term or long-term depending on the assessment scope. Medium-term is particularly relevant to corporations and nations, and timescales of 5–10 years, whereas long-term is for global assessments and timescales of decades or longer. Medium- and long-term supply risk includes a geological, technological, and economic (gte) indicator, which comprises depletion time (dt) and companion metal fraction (cf) factors. Depletion time represents several combined effects: reserves; mining production; demand; output from the use phase; quantity landfilled; secondary (scrap) supply; net loss to tailings, slag, and other by-products; lifetime; and end-of-life recycling rate. Medium-term supply risk additionally comprises a social and regulatory (s_r) indicator, containing policy potential index (ppi) and human development index (hdi) factors, and a geopolitical (gp) indicator, containing worldwide governance indicators – political stability & absence of violence/terrorism (wgi_pv), and global supply concentration (gsc) factors.

Environmental implications represent the potential burdens that materials place on the environment throughout their (life) cycles, e.g., damage to ecosystems caused by toxic emissions from metal production, which may limit their availability. The environmental implications indicator for a material is determined by grouping damage to human health and to ecosystems, which are produced through a cradle-to-gate life cycle assessment for that material. Environmental implications data in YSTAFDB utilize a functional unit of 1 kg material at the factory gate, inventory data from Ecoinvent 2.2^30^, and the ReCiPe v1.10 method with world normalization and hierarchist weighting^31^.

Vulnerability to supply restriction characterizes the importance of a material to society, e.g., iron (Fe) is globally relied upon in infrastructure, housing, vehicles, etc., so is relatively important. It is determined differently on corporate, national, and global levels. Although included in the criticality methodology developed by Graedel and colleagues, values at the corporate level were not calculated; readers are directed to^5,22^ for methodological details at this level. National level vulnerability to supply restriction contains an importance (i) indicator comprised of material assets (ma) and national economic importance (ne) factors, a substitutability (s) indicator comprised of substitute performance (sp), substitute availability (sa), environmental impact ratio (er), and net import reliance ratio (irr) factors, and a susceptibility (su) indicator comprised of global innovation index (gii) and net import reliance (ir) factors. Global vulnerability to supply restriction contains an importance (i) indicator comprised of a single material assets (ma) factor, and a substitutability (s) indicator, comprised of substitute performance (sp), substitute availability (sa), and environmental impact ratio (er) factors.

### Recycling

Material cycles also characterize material recycling. Recycling related properties of material cycles reported by Graedel and colleagues were summarized in a few key publications^6,14,32^. These properties include:

1. in-use dissipation, indicating unrecoverable material lost during use;
2. rates of currently unrecyclable and potentially recyclable material, and of end-of-life recycling;
3. market shares of key material applications, such as construction, machinery, packaging, etc.; and
4. unspecified recycling potential, which is used in the absence of data.

**Fig. 5:**
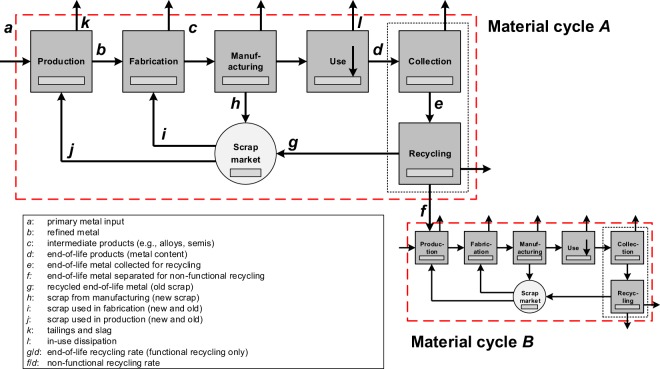
Various recycling related characteristics of material cycles. Adapted from^6^.

Currently unrecyclable material is material that is prevented from recycling due to prevailing technological and economic barriers. Potentially recyclable material may be functionally recycled, non-functionally recycled, or not recovered. End-of-life recycling rates in YSTAFDB correspond to functionally recycled material only, i.e., percentages of material sent back to production, fabrication, etc. (in the same material cycle) from waste management relative to the amount of material sent to waste management from use at end-of-life. Various recycling related characteristics of material cycles are shown in Fig. 5.

### Data input and templates

Numerous differently formatted STAF data were input into YSTAFDB. Many of these data exist graphically and in portable document format (pdf), which therefore required significant manual effort to extract. Some data were present in spreadsheet format: these data were typically sorted by system boundary properties (reference material, reference timeframe, reference space), and by process or flow name. We consistently reformatted STAF data from material cycles into uniformly formatted spreadsheets, hereafter ‘templates’, which were then consistently parsed in a later step. The template structure was specified to allow sufficient annotation of key STAF data properties, e.g., material name, units, etc. We included these key properties in YSTAFDB as metadata. STAF data for a single material cycle publication were used to fill one template, such that ~60 filled templates were used to develop the material cycles database tables in YSTAFDB. An example template is provided along with this contribution^33^.

## Data Records

**Fig. 6:**
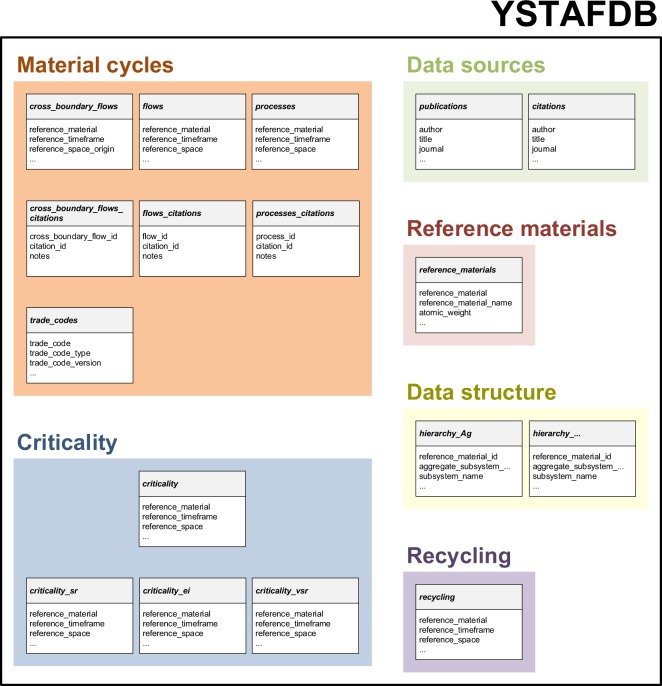
Illustration of the core and supplementary tables in YSTAFDB. YSTAFDB tables are represented by grey and white boxes; table headings are shown as bold italic text on grey backgrounds, and categories in data records are shown as normal text on white backgrounds. Tables are grouped by type and not all *hierarchy* tables are shown, for reader convenience only. Table groups are represented by color shaded regions and group headings are in large bold colored font.

YSTAFDB contains the fifteen core tables shown in Table 1 and Fig. 6, which are each provided as csv files^33^. Headings of tables in YSTAFDB are hereafter written in italics. A complete list of core tables and fields in YSTAFDB, including descriptions and examples, can be viewed in Table S1 of the Supplementary Information. These tables are supplemented by 63 *hierarchy* tables (Table 1 and Fig. 6), which are also each provided as csv files^33^. Each *hierarchy* table represents the complete process/subsystem hierarchy of a reference material cycle for which data are available in YSTAFDB. Excerpts from the *flows* data table and *flows_citations* mapping table are shown in Tables 2 and 3, respectively, to indicate their nature. YSTAFDB contains a total of 115,829 data records in the *flows*, *cross_boundary_flows*, *processes*, *recycling*, *criticality*, *criticality_sr*, *criticality_ei*, and *criticality_vsr* data tables.

**Table 1:** Descriptions of the core and supplementary tables in YSTAFDB.

Name	Description	Example^a^
***Core tables***		
*citations*	Citations for data sources that were used to determine data reported in published studies and stored in YSTAFDB	Brunner, P. H., “Urban Mining A Contribution to Reindustrializing the City”, J. Ind. Ecol., 15, 339–341 (2011).
*criticality*	Overall criticality data	23.2
*criticality_ei*	Environmental implications criticality data	2.73
*criticality_sr*	Supply risk criticality data	47.8
*criticality_vsr*	Vulnerability to supply restriction criticality data	41.7
*cross_boundary_flows*	Flows that intersect reference spaces, e.g., international trade flows	3.4 Gg
*cross_boundary_flows_citations*	Mapping of rows in cross_boundary_flows to corresponding rows in citations	200009344, 78^b^
*flows*	Flows that do not intersect reference spaces, e.g., domestic transport	6.5 Mg
*flows_citations*	Mapping of rows in flows to corresponding rows in citations	200002209, 28^b^
*processes*	Information for transformation, distribution, and storage of materials, including stocks	86.2 Gg^c^
*processes_citations*	Mapping of rows in processes to corresponding rows in citations	200001616, 30^b^
*publications*	Citations for published studies that contain the data stored in YSTAFDB	Graedel, T. E., Cao, J., “Metal Spectra as Indicators of Development”, Proc. Natl. Acad. Sci. USA 107(49), 20905–20910 (2010).
*recycling*	Recycling metrics for materials and substances	55.2%
*reference_materials*	Materials that are used as references for applying mass conservation	Ag, Silver, 1
*trade_codes*	Characteristics of trade data classification systems	281700
***Supplementary tables*** ^d^		
*hierarchy_Ag*	Process tree and labeling notation for data records in the *processes*, *flows*, and *cross_boundary_flows* tables, specified using the UMIS data structure	1, F&M, products, 2;1′;1

^a^An example represents an individual data record in a YSTAFDB table. ^b^There are two main data entries in mapping tables, which each correspond to a data record in another YSTAFDB table. The names of the corresponding tables (e.g., *cross_boundary_flows* and *citations*) occur in the name of the mapping table (e.g., *cross_boundary_flows_citations*). ^c^Such quantitative values and units in the processes table refer to quantities of stocks. ^d^Only a single *hierarchy* table is shown here for reader convenience; YSTAFDB contains 63 equivalently formatted *hierarchy* tables.

**Table 2:** An excerpt from the *flows* table in YSTAFDB. ‘\N’ indicates a ‘null’ data entry (i.e., an empty cell).

Reference_material	Reference_timeframe	Reference_space	…	Flow_label	…	Quantity	Quantity_unit	…	Publication_id	Flow_id
…	…	…	…	…	…	…	…	…	…	…
Ag (Silver)	1997	India	…	1.ENV.5;1.D.12;12_1.PEM.1.T.1;1	…	62	Mg	…	15	1500000003
Ag (Silver)	1997	Indonesia	…	1.ENV.5;1.D.12;12_1.PEM.1.T.1;1	…	315	Mg	…	15	1500000004
Ag (Silver)	1997	Japan	…	1.ENV.5;1.D.12;12_1.PEM.1.T.1;1	…	109	Mg	…	15	1500000005
Ag (Silver)	1997	Malaysia	…	1.ENV.5;1.D.12;12_1.PEM.1.T.1;1	…	12	Mg	…	15	1500000006
…	…	…	…	…	…	…	…	…	…	…
Ag (Silver)	1997	Earth and orbital bodies	…	1.PEM.1.T.1;1_1.ENV.5;1.T.23;23	…	0.2	Gg	…	17	1700000594
Ag (Silver)	1997	Earth and orbital bodies	…	1.PEM.1.T.1;1_1.F&M.2.T.1;1	…	29	Gg	…	17	1700000602
Ag (Silver)	1997	Earth and orbital bodies	…	1.F&M.2.D.2;2_1.ENV.5;1.T.23;23	…	0.15	Gg	…	17	1700000618
…	…	…	…	…	…	…	…	…	…	…
Cu (Copper)	1994	Greece	…	13.USE.3;1′;1;1.D.4;4_13.WMR.4.T.1;1	…	1799.2	Gg	…	19	1900000027
Cu (Copper)	1994	Finland	…	13.USE.3;1′;1;1.D.4;4_13.WMR.4.T.1;1	…	1351.5	Gg	…	19	1900000028
…	…	…	…	…	…	…	…	…	…	…
Fe (Iron)	2001	United States of America	…	17.WMR.4;1.D.2;2_17.PEM.1;1;2.T.3;3	…	8.44	Tg	…	51	25226
Fe (Iron)	2002	United States of America	…	17.WMR.4;1.D.2;2_17.PEM.1;1;2.T.3;3	…	7.97	Tg	…	51	25227
Stainless steel (General stainless steel alloy)	2000	Germany	…	99.WMR.4;1;2.D.4;4_99.PEM.1;1.T.3;3	…	397.606	Gg	…	59	25228
…	…	…	…	…	…	…	…	…	…	…

**Table 3:** An excerpt from the *flows_citations* table in YSTAFDB. ‘\N’ indicates a ‘null’ data entry (i.e., an empty cell).

Flow_id	Citation_id	Notes
200000199	48	\N
200000200	48	\N
200000201	48	\N
200000202	48	\N
200000203	48	\N
200000204	48	\N
200000205	48	\N
200000206	48	\N
200000207	48	\N
200000208	48	\N
200000209	48	\N
200000210	48	\N
200000211	48	\N
200000212	48	\N
200000213	48	\N
200000214	48	\N
200000215	48	\N
200000216	48	\N
200000217	48	\N
200000218	48	\N
200000219	48	\N
200000220	48	\N
…	…	…

### Publications and citations

Material cycles, recycling, and criticality data from 60 published studies were stored in YSTAFDB. These data were themselves produced through the collection, interpretation, and analysis of data from peer-reviewed journal papers^34^, government reports^35^, metal association and study group reports^36^, industry consultations, and other sources^28,37^. The sources of these data are defined as *citations* in YSTAFDB. The published studies that analyzed these data are referred to as *publications* in YSTAFDB (and available in the *publications* table). Information such as author, title, journal, year, doi, etc. are recorded in these tables, as well as unique identifiers (hereafter ‘ids’) for each data record. Data records in the *publications* and *citations* tables are referred to using their ids in other YSTAFDB tables.

### Material cycles

The *cross_boundary_flows*, *flows*, and *processes* tables contain most of the data records in YSTAFDB. Data records in these tables comprise:

1. system boundary properties, i.e., reference material (e.g., iron [Fe]), reference space (e.g. Australia), and reference time (e.g., 2010);
2. material cycle location, i.e., relationship of data record to the system boundary (e.g., inside), aggregate subsystem module (e.g., fabrication and manufacturing), subsystem (e.g., production), and UMIS label (e.g., *1*.*ENV*.*5;1*.*D*.*12;12*_*1*.*PEM*.*1*.*T*.*1*;*1*)^23^ such that each data record is indexed to a position in a material (life) cycle;
3. quantitative data and their units (e.g., 1672 Mg), their concentrations and associated units (e.g. 0.5 mass fraction), their uncertainty values and associated units and types (e.g., 40% plus-minus), their reliability (e.g., good), and quantitative residuals (often termed ‘phantom flows’) and their concentrations from mass or energy balances (e.g., 28 Gg with a concentration of 1 mass fraction);
4. core metadata related to processes, flows, and cross boundary flows such as names of materials (e.g., mining output) and processes (e.g., alumina refining), and types of processes (e.g., transformative) and stock (e.g., net added); and
5. peripheral metadata describing the methods used to produce the data (e.g., estimated), the publication id from which the data records were derived (e.g., 8), our notes about the data records, and unique ids for each data record.

Reference materials and their ids are related explicitly in the *reference_materials* table. The reference material for a data record is shown in the first column in the *cross_boundary_flows*, *flows*, and *processes* tables in name form (e.g., Zn [Zinc]), but as the first component of process and flow labels in id form (e.g., ‘*58*’ in process label *58*.*USE*.*3*.*T*.*1*;*1*). Reference materials are also specified in id form in *hierarchy* tables (e.g., hierarchy_Zn.csv)^33^.

Data records in *hierarchy* tables relate directly to process and flow labels, which explicitly indicate the process/subsystem hierarchy for each reference material cycle, including occurrences of consistent and divergent disaggregation: the absence of an apostrophe indicates consistent disaggregation; the presence of an apostrophe indicates divergent disaggregation; and a semi-colon in the subsystem code indicates an aggregation/disaggregation step up or down the process/subsystem hierarchy. A process number indicates the unique location of a process within a subsystem; process codes are derived from process numbers by stripping apostrophes (instances of divergent disaggregation) and then representing the numbers as coordinates along the (square) matrix diagonal of a matrix-type UMIS diagram, where each transformative process in UMIS (and this matrix) is succeeded immediately by a distributive process. Therefore, process numbers map to odd integers in transformative process codes, and even integers in distributive process codes. This derivation of process codes applies to process numbers that both contain apostrophes, and do not contain apostrophes, and is shown explicitly in the *hierarchy* tables^33^. Semi-colons in process codes are used to separate the numerical values for clarity, e.g., a process number of *3*′ maps to process codes of *5*;*5* and *6*;*6*. Aggregate subsystem modules and their abbreviations (e.g., ‘*USE*’ in process label *58*.*USE*.*3*.*T*.*1*;*1*) are specified in *hierarchy* tables at the most aggregated subsystem level (i.e., at the one-digit subsystem code level), which here correspond to material cycles stages.

A *trade_codes* table is also included in YSTAFDB; it comprises metadata specifically associated with cross boundary flows that represent international trade (e.g., HS classification 8415). Three mapping tables, *cross_boundary_flows_citations*, *flows_citations*, and *processes_citations*, are included in YSTAFDB to relate data records in the *cross_boundary_flows*, *flows*, and *processes* tables to one or more data records in the *citations* table. They are necessary to fully describe data records that were determined using data from multiple sources.

### Recycling

The *recycling* table in YSTAFDB contains data records that specifically describe recycling characteristics of material cycles. We distinguish the *recycling* table from the *cross_boundary_flows*, *flows*, and *processes* tables for the following three key reasons:

1. to facilitate simpler searching of recycling data in YSTAFDB;
2. because recycling indicators are relatively well-defined, e.g., the end of life recycling ratio, the need to describe them using a data structure like UMIS^23^ is reduced; and
3. because we interpret them as results from analyses of material cycles data. We believe that this is advantageous in adding, updating, and using recycling data records in YSTAFDB.

Data records in the *recycling* table comprise:

1. system boundary properties (e.g., reference timeframe, reference space, etc.);
2. core metadata (e.g. process name, material name);
3. quantitative data and their units (e.g., 10%), their uncertainty values and associated units and type (e.g., 25% plus-minus), and their reliabilities (e.g., good);
4. peripheral metadata describing the methods used to produce the data (e.g., estimated), the publication id from which the data records were derived (e.g., 60), notes about the data records, and unique ids for each data record.

Recycling data in YSTAFDB are classified by recycling type, e.g., potentially recyclable, unrecyclable, etc. They may also be classified by recycling rates, such as the end of life recycling rate. Therefore, each data record in the *recycling* table is additionally described by its type of recycling and use (e.g. currently unrecyclable, market share, end of life recycling rate, etc.).

### Criticality

Criticality data are stored in a similar manner to recycling data in YSTAFDB, that is, distinct from material cycles data (*processes*, *flows*, *cross_boundary_flows* tables). ‘Overall’ criticality data are stored in the *criticality* table. These data represent combinations of supply risk, environmental implications, and vulnerability to supply restriction indicators, consistent with the criticality methodology developed by Graedel and colleagues^5,22^. Data for these three indicators are stored in their own tables, *criticality_sr* (supply risk), *criticality_ei* (environmental implications), and *criticality_vsr* (vulnerability to supply restriction), respectively. These tables contain data for more specific criticality indicators, e.g., depletion time (dt), material assets (ma), and global innovation index (gii). A complete list of specific criticality indicators is provided in^22^.

Data records in the *criticality*, *criticality_sr*, *criticality_ei*, and *criticality_vsr* tables refer to the timeframes of the criticality assessments, e.g., medium-term or long-term. These timeframes, the methods used to produce the data, notes about the data records, and system boundary properties (reference material, reference timeframe, reference space), are also stored in these criticality related tables.

## Technical Validation

Most data records in YSTAFDB went through the standard publication process in peer reviewed academic journals prior to this data release. Many of these data were entered directly into YSTAFDB without modification. The data were manually extracted from text, tables, and figures in the main text of papers, supporting information pdf files, and spreadsheets. Some data were taken from reports and additional data files provided by personal communication with corresponding authors, which may or may not have gone through a peer review process. These data are entered into YSTAFDB ‘as is’. Exceptionally, some material cycle data were recalculated for consistency with their system contexts recorded in YSTAFDB. This recalculation procedure involved locating STAF data within new material cycle contexts, assigning UMIS process and flow labels to these data, and then performing unsteady state mass balances around processes in the material cycles considering all of the relevant (re-specified) STAF data. This procedure was necessary to unambiguously define some STAF data in YSTAFDB. Explanatory notes were added to data records in instances where recalculation was performed.

## Usage Notes

This paper is accompanied by fifteen core csv files, each of which corresponds to a table in YSTAFDB^33^. They are supplemented by 63 *hierarchy* tables in csv file format (e.g., hierarchy_Al.csv), one for each reference material cycle recorded in YSTAFDB^33^. These *hierarchy* tables contain descriptions of the appropriate process/subsystem hierarchies in UMIS label format and nomenclature^23^. These csv files and tables comprise YSTAFDB (Table 1 and Fig. 6)^33^. The individual csv files are described in the *Data Records* section. An alternative version of YSTAFDB, comprising the same data, but as a set of 38 tables in csv file, MySQL, and PostgreSQL formats, is available through a USGS data release on ScienceBase^38^. Complete details of this alternative version are provided on ScienceBase.

We provide the following three examples to demonstrate the utility of YSTAFDB:

1. Searching for the quantity of aluminum (Al) in the in-use stock of transport products in 1990. This search may be accomplished by opening the *processes* table and filtering for ‘Al’ in the reference_material column, ‘1990’ in the reference_timeframe column, ‘use’ in the aggregate_subsystem_module column, ‘transport’ in the process_name column, and ‘total’ in the stock_type column. This search results in a single data record showing that the total mass of Al in transport products in the in-use stock in the United States in year 1990 was 18,212,632 Mg, and that these data were published in Chen *et al*.^39^ Searching through Chen *et al*.^39^, the data can be located in Figure 10a, which is a result from this dynamic MFA study.
2. Searching for flows in the iridium (Ir) cycle. This search may be accomplished by opening the *flows* table and filtering for ‘Ir’ in the reference_material column. This search results in nine data records that describe flows of Ir along its cycle, from production to waste management. For example, the data record with a (unique) flow_id of 5462 shows that 1.3 Mg of ‘industrial output’ materials were output from a process in the waste management and recycling stage of the global Ir cycle in year 2010; the corresponding *hierarchy_Ir* table indicates on row 35 that this material originated from an ‘industrial’ process, and also that this ‘industrial’ process is a child of the waste, waste management, and aggregate waste management subsystems, i.e., the ‘industrial’ process is an industrial waste management process. The user can search through the corresponding publication^40^ to observe that this data record originates from Figure 7.2, which presents its dynamic MFA results as losses from the global anthropogenic Ir cycle without explicitly indicating destination processes for these flows.
3. Searching for the recycling rate of boron (B) containing products. This search may be accomplished by opening the *recycling* table and filtering for ‘B’ in the reference_material column. This search results in one data record that quantifies the global end of life recycling rate of B in all end of life products as 0.5 ± 0.5%, and that this value is representative of the timeframe from year 2000 to year 2005. The user can search through the corresponding publication^32^ to find that this data record originates from Table F1 (pg. 36), which contains an end of life recycling rate of 0–1% for B.

## Supplementary Information

### ISA-Tab metadata file

Download metadata file

### Supplementary information

Supplementary Information

## Data Availability

Each filled template file was converted into csv format and then parsed using a Python script titled ‘templates_0.2’^33^. This script converts STAF data in template files into csv files with the same table format as present in data and mapping tables in YSTAFDB. The Python script produces the following csv files: *cross_boundary_flows*, *cross_boundary_flows_citations*, *flows*, *flows_citations*, *processes*, *processes_citations*. Therefore, this set of six csv files were produced ~60 times to develop the material cycles part of YSTAFDB, one for each filled template (i.e., for each material cycle publication). All csv files of the same type, e.g., *cross_boundary_flows*, were then merged using another Python script titled ‘merged_1.0’^33^. This procedure yielded six merged csv files, one for each type: *cross_boundary_flows*, *cross_boundary_flows_citations*, *flows*, *flows_citations*, *processes*, and *processes_citations*. Some manual cleaning was then performed to correct any formatting errors identified. All other tables in YSTAFDB were manually produced directly from publications, including *criticality*, *recycling*, and their related tables.
