# Supplementary material for: YSTAFDB, a unified database of material stocks and flows for sustainability science
Source: Sci Data. 2019 Jun 7;6:84. doi: 10.1038/s41597-019-0085-7 (PMC6555823; doi:10.1038/s41597-019-0085-7)
Supplement: Supplementary file 2 — Supplementary Information [file 41597_2019_85_MOESM2_ESM.pdf]

Supplementary information for:

YSTAFDB, a unified database of material stocks and flows for sustainability science

Rupert J. Myers [1,2,a,*], Barbara K. Reck [2,b], T. E. Graedel [2,c]

[1] Current address: School of Engineering, The University of Edinburgh, King's Buildings, Sanderson Building, Edinburgh, EH9 3BF, United Kingdom

[2] School of Forestry & Environmental Studies, Yale University, 195 Prospect St, New Haven 06511 Connecticut, United States

[*] Corresponding author. Email: [a] rupert.myers@ed.ac.uk; [b] barbara.reck@yale.edu; [c] thomas.graedel@yale.edu.

Table of Contents

Tables and fields in YSTAFDB S2

Additional discussion of the material cycles data in YSTAFDB S13

Duplicated information S13

Hierarchy tables S13

References in this supplementary information document S14

Tables and fields in YSTAFDB

Table S1 provides a complete list of core tables and fields in YSTAFDB, as well as their descriptions and some representative values. Table S1 complements Table 1 in the main text of the paper.

Table S1. Descriptions of the core tables in YSTAFDB (continues overleaf).

Table	Field	Description	Example
citations	author	author(s) of the data record	Spatari S.; Bertram M.; Fuse K.; Graedel T.E.; Shelov E.
citations	title	title of the data record	"The contemporary European zinc cycle: 1-year stocks and flows"
citations	journal	journal of the data record	Res. Conserv. Recyc.
citations	volume	volume of the journal	39
citations	number	number/issue of the journal	2
citations	pages	page numbers of the data record	137-160
citations	year	year that the data record was published	2003
citations	doi	doi of the data record	\N
citations	URL	URL of the data record	http://dx.doi.org/10.1016/S0921-3449(02)00168-4
citations	eprint	eprint of the data record	\N
citations	notes	notes related to the data record	\N
citations	citation_id	unique integer id of the citations data record	48
criticality	reference_material	reference material (system boundary property)	Ru (Ruthenium)
criticality	reference_timeframe	reference timeframe (system boundary property)	2008
criticality	reference_space	reference space (system boundary property)	Global
criticality	criticality_timeframe	time perspective of the criticality assessment	long-term
criticality	criticality_magnitude_normalized	radius of the overall criticality vector	49.81818174
criticality	criticality_sr_angle	angle from the supply risk axis of the overall criticality vector	56.45586552
criticality	criticality_ei_angle	angle from the environmental implications axis of the overall criticality vector	51.10994708
criticality	criticality_vsr_angle	angle from the vulnerability to supply risk axis of the overall criticality vector	56.75856686
criticality	criticality_sr	supply risk value	124
criticality	criticality_ei	environmental implications value	25
criticality	criticality_vsr	vulnerability to supply restriction value	124

criticality	method	methodology used to determine the data record	\N
criticality	publication_id	unique integer id of the publications data record	35
criticality	notes	notes related to the data record	Angles are measure in degrees (0-180) from the Supply Risk (sr); Environmental Implications (ei); and Vulnerability to Supply Restriction (vsr) axes
criticality	criticality_id	unique integer id of the criticality data record	25
criticality_ei	reference_material	reference material (system boundary property)	Pr (Praseodymium)
criticality_ei	reference_timeframe	reference timeframe (system boundary property)	2008
criticality_ei	reference_space	reference space (system boundary property)	All spatial boundaries
criticality_ei	criticality_timeframe	time perspective of the criticality assessment	\N
criticality_ei	ei	environmental implications value	6.452654234
criticality_ei	method	methodology used to determine the data record	\N
criticality_ei	publication_id	unique integer id of the publications data record	35
criticality_ei	notes	notes related to the data record	\N
criticality_ei	criticality_ei_id	unique integer id of the criticality_ei data record	37
criticality_sr	reference_material	reference material (system boundary property)	AgInCd alloy (80% Silver mining production; 15% 80% Zinc mining production; 15% Tin mining production; 5% Copper mining production; 5% Zinc mining production)
criticality_sr	reference_timeframe	reference timeframe (system boundary property)	2008

criticality_sr	reference_space	reference space (system boundary property)	National or corporation
criticality_sr	criticality_timeframe	time perspective of the criticality assessment	medium-term
criticality_sr	sr	supply risk value	69.03753707
criticality_sr	gte	geological, technological, and economic value	84.31054465
criticality_sr	dt	depletion time value	91.9072
criticality_sr	cf	companion metal fraction value	76.71388929
criticality_sr	s_r	social and regulatory value	62.02325831
criticality_sr	ppi	policy potential index value	48.14212036
criticality_sr	hdi	human development index value	75.90439625
criticality_sr	gp	geopolitical value	60.77880825
criticality_sr	wgi_pv	worldwide governance indicators - political stability & absence of violence/terrorism value	58.613181
criticality_sr	gsc	global supply concentration value	62.9444355
criticality_sr	method	methodology used to determine the data record	\N
criticality_sr	publication_id	unique integer id of the publications data record	35
criticality_sr	notes	notes related to the data record	\N
criticality_sr	criticality_sr_id	unique integer id of the criticality_sr data record	67
criticality_vsr	reference_material	reference material (system boundary property)	Sb (Antimony)
criticality_vsr	reference_timeframe	reference timeframe (system boundary property)	2008
criticality_vsr	reference_space	reference space (system boundary property)	United States of America
criticality_vsr	criticality_timeframe	time perspective of the criticality assessment	medium-term
criticality_vsr	vsr	vulnerability to supply restriction value	49.06047631
criticality_vsr	i	importance value	50.523988
criticality_vsr	ma	material assets value	100
criticality_vsr	ne	national economic importance value	1.047976006
criticality_vsr	s	substitutability value	39.32410759
criticality_vsr	sp	substitute performance value	58.875
criticality_vsr	sa	substitute availability value	45.65899381
criticality_vsr	er	environmental impact ratio value	14.0066226

criticality_vsr	irr	net import reliance ratio value	38.75581395
criticality_vsr	su	susceptibility value	57.33333333
criticality_vsr	ir	global innovation index value	86
criticality_vsr	gii	net import reliance value	28.66666667
criticality_vsr	method	methodology used to determine the data record	\N
criticality_vsr	publication_id	unique integer id of the publications data record	35
criticality_vsr	notes	notes related to the data record	\N
criticality_vsr	criticality_vsr_id	unique integer id of the criticality_vsr data record	32
cross_boundary_flows	reference_material	reference material (system boundary property)	Zn (Zinc)
cross_boundary_flows	reference_timeframe	reference timeframe (system boundary property)	2010
cross_boundary_flows	reference_space_origin	reference space of the origin process (system boundary property)	Global
cross_boundary_flows	reference_space_destination	reference space of the destination process (system boundary property)	India
cross_boundary_flows	system_boundary_origin	location of the origin process with respect to the cross_boundary_flow system boundary	inside
cross_boundary_flows	system_boundary_destination	location of the destination process with respect to the cross_boundary_flow system boundary	inside
cross_boundary_flows	aggregate_subsystem_module_origin	aggregate subsystem module containing the origin process in the reference material cycle	fabrication and manufacturing (F&M)
cross_boundary_flows	aggregate_subsystem_module_destination	aggregate subsystem module containing the destination process in the reference material cycle	fabrication and manufacturing (F&M)
cross_boundary_flows	subsystem_name_origin	subsystem containing the origin process in the reference material cycle	galvanizing
cross_boundary_flows	subsystem_name_destination	subsystem containing the destination process in the reference material cycle	galvanizing
cross_boundary_flows	cross_boundary_flow_label	UMIS label of the cross boundary flow (indicating its origin process and destination process)	58.F&M.2;1;1';1'.D.2;2_58.F&M.2;1;1';1'.D.2;2
cross_boundary_flows	material_name	name of material in the cross boundary flow	galvanizing product output

cross_boundary_flows	cross_boundary_flow_type	type of cross boundary flow associated with the data record (e.g., import, export, net import)	import
cross_boundary_flows	trade_code_id	unique integer id of the trade_code data record	7
cross_boundary_flows	trade_data_source_id	source of the trade data (supplier or consumer)	\N
cross_boundary_flows	quantity	quantity of the data record (of a reference material)	55.060742
cross_boundary_flows	quantity_unit	unit of quantity associated with the data record	Gg
cross_boundary_flows	concentration	concentration of the data record (of a reference material in a product)	0.04
cross_boundary_flows	concentration_unit	unit of concentration associated with the data record	mass fraction (w/w)
cross_boundary_flows	uncertainty	uncertainty of the data record	\N
cross_boundary_flows	uncertainty_unit	unit of uncertainty associated with the data record	\N
cross_boundary_flows	uncertainty_type	type of uncertainty associated with the data record	\N
cross_boundary_flows	reliability	reliability of the data record	\N
cross_boundary_flows	method	methodology used to determine the data record	\N
cross_boundary_flows	publication_id	unique integer id of the publications data record	2
cross_boundary_flows	notes	notes related to the data record	\N
cross_boundary_flows	cross_boundary_flow_id	unique integer id of the cross_boundary_flows data record	200012590
cross_boundary_flows_citations	cross_boundary_flow_id	unique integer id of the cross_boundary_flows data record	200014468
cross_boundary_flows_citations	citation_id	unique integer id of the citations data record	65
cross_boundary_flows_citations	notes	notes related to the data record	\N
flows	reference_material	reference material (system boundary property)	Pb (Lead)
flows	reference_timeframe	reference timeframe (system boundary property)	2004
flows	reference_space	reference space (system boundary property)	China

flows	system_boundary_origin	location of the origin process with respect to the flows system boundary	inside
flows	system_boundary_destination	location of the destination process with respect to the flows system boundary	inside
flows	aggregate_subsystem_module_origin	aggregate subsystem module containing the origin process in the reference material cycle	production of engineering materials (PEM)
flows	aggregate_subsystem_module_destination	aggregate subsystem module containing the destination process in the reference material cycle	production of engineering materials (PEM)
flows	subsystem_name_origin	subsystem containing the origin process in the reference material cycle	mining beneficiation
flows	subsystem_name_destination	subsystem containing the destination process in the reference material cycle	mining beneficiation
flows	flow_label	UMIS label of the flow (indicating its origin process and destination process)	35.PEM.1;1;1;1.T.3;3_35.PEM.1;1;1;1.D.4;4
flows	material_name	name of material in the flow	tailings output
flows	quantity	quantity of the data record (of a reference material)	146
flows	quantity_unit	unit of quantity associated with the data record	Gg
flows	concentration	concentration of the data record (of a reference material in a product)	1
flows	concentration_unit	unit of concentration associated with the data record	mass fraction (w/w)
flows	uncertainty	uncertainty of the data record	\N
flows	uncertainty_unit	unit of uncertainty associated with the data record	\N
flows	uncertainty_type	type of uncertainty associated with the data record	\N
flows	reliability	reliability of the data record	poor (P)
flows	method	methodology used to determine the data record	\N
flows	publication_id	unique integer id of the publications data record	11
flows	notes	notes related to the data record	\N
flows	flow_id	unique integer id of the flows data record	11180
flows_citations	flow_id	unique integer id of the flows data record	200000739

flows_citations	citation_id	unique integer id of the citations data record	15
flows_citations	notes	notes related to the data record	\N
processes	reference_material	reference material (system boundary property)	Fe (Iron)
processes	reference_timeframe	reference timeframe (system boundary property)	2000
processes	reference_space	reference space (system boundary property)	United States of America; Connecticut
processes	system_boundary	location of the process with respect to the system boundary	inside
processes	aggregate_subsystem_module	aggregate subsystem module containing the process in the reference material cycle	use (USE)
processes	subsystem_name	subsystem containing the process in the reference material cycle	rail
processes	process_label	UMIS label of the process	17.USE.3;1;4;6.T.3;3
processes	process_type	type of process (transformative, distributive, storage)	transformative
processes	process_name	name of process	overhead contact system
processes	stock_type	type of stock in the process (total stock "total", net added to stock "net added", deposited to stock "deposited", withdrawn from stock "withdrawn")	total
processes	stock_quantity	quantity of stock in the process (of a reference material)	31
processes	stock_quantity_unit	unit of stock quantity associated with the data record	Gg
processes	stock_concentration	concentration of stock in the process (of a reference material in a product)	1
processes	stock_concentration_unit	unit of stock concentration associated with the data record	mass fraction (w/w)
processes	stock_uncertainty	uncertainty of the data record	\N
processes	stock_uncertainty_unit	unit of uncertainty associated with the data record	\N
processes	stock_uncertainty_type	type of uncertainty associated with the data record	\N
processes	stock_reliability	reliability of the data record	adequate (A)
processes	residual_quantity	quantity of residual stock in the process (of a reference material, which is a	\N

		residual from a mass balance around the process)	
processes	residual_quantity_unit	unit of residual stock quantity associated with the data record	\N
processes	residual_concentration	concentration of residual stock in the process (of a reference material in a product)	\N
processes	residual_concentration_unit	unit of residual stock concentration associated with the data record	\N
processes	method	methodology used to determine the data record	\N
processes	publication_id	unique integer id of the publications data record	50
processes	notes	notes related to the data record	\N
processes	process_id	unique integer id of the processes data record	2266
processes_citations	process_id	unique integer id of the processes data record	200001587
processes_citations	citation_id	unique integer id of the citations data record	30
processes_citations	notes	notes related to the data record	\N
publications	author	author(s) of the data record	Johnson J.; Jirikowic J.; Bertram M.; van Beers D.; Gordon R.B.; Henderson K.; Klee R.J.; Lanzano T.; Lifset R.; Oetjen L.; Graedel T.E.
publications	title	title of the data record	"Contemporary Anthropogenic Silver Cycle: A Multilevel Analysis"
publications	journal	journal of the data record	Environ. Sci. Technol.
publications	volume	volume of the journal	39
publications	number	number/issue of the journal	\N
publications	pages	page numbers of the data record	4655-4665
publications	year	year that the data record was published	2005
publications	doi	doi of the data record	10.1021/es048319x
publications	URL	URL of the data record	http://dx.doi.org/10.1021/es048319x
publications	eprint	eprint of the data record	\N

publications	notes	notes related to the data record	SI available
publications	publication_id	unique integer id of the publications data record	12
recycling	reference_material	reference material (system boundary property)	Ru (Ruthenium)
recycling	reference_timeframe	reference timeframe (system boundary property)	2008
recycling	reference_space	reference space (system boundary property)	Global
recycling	process_name	process related to the data record	chemical
recycling	material_name	material related to the data record	metal; oxides
recycling	recycling_use_type	type of recycling related property describing the data record	currently unrecyclable
recycling	quantity	quantity of the data record	1.1
recycling	quantity_unit	unit of quantity associated with the data record	%
recycling	uncertainty	uncertainty of the data record	\N
recycling	uncertainty_unit	unit of uncertainty associated with the data record	\N
recycling	uncertainty_type	type of uncertainty associated with the data record	\N
recycling	reliability	reliability of the data record	\N
recycling	method	methodology used to determine the data record	reported
recycling	publication_id	unique integer id of the publications data record	60
recycling	notes	notes related to the data record	percent of total market share
recycling	recycling_id	unique integer id of the recycling data record	696
reference_materials	reference_material	short hand name of the reference material	Te
reference_materials	reference_material_name	name of the reference material	Tellurium
reference_materials	atomic_weight	atomic weight of the reference material (g/mol)	128
reference_materials	notes	notes related to the data record	\N
reference_materials	reference_material_id	unique integer id of the reference_material data record	50
trade_codes	trade_code	trade code related to the data record	721030

trade_codes	trade_code_type	type of trade code related to the data record (classification system, e.g., harmonised system)	hs
trade_codes	trade_code_version	version of the trade code type related to the data record	\N
trade_codes	commodity	commodity that the data record describes (i.e., a product)	\N
trade_codes	description	description of the commodity that the data record describes	Flat-rolled products of iron-non-alloy steel; of a width of 600mm-more; electrolytically plated-coated with zinc
trade_codes	publication_id	unique integer id of the publications data record	6
trade_codes	notes	notes related to the data record	\N
trade_codes	trade_code_id	unique integer id of the trade_codes data record	7

Additional discussion of the material cycles data in YSTAFDB

Duplicated information

Several fields in the *cross_boundary_flows*, *flows*, and *processes* tables were included to simplify manual querying and readability of YSTAFDB, however, this information can also be obtained from unified materials information system (UMIS)[1] labels and *hierarchy* tables. Information in the cross_boundary_flow_label, flow_label, and process_label fields in these *cross_boundary_flows*, *flows*, and *processes* tables, and in all fields in the *hierarchy* tables, can be used to specify the following fields in the *cross_boundary_flows*, *flows*, and *processes* tables:

i. reference_material;
ii. aggregate_subsystem_module;
iii. aggregate_subsystem_module_origin;
iv. aggregate_subsystem_module_destination;
v. subsystem_name;
vi. subsystem_name_origin;
vii. subsystem_name_destination;
viii. process_type; and
ix. process_name.

Hierarchy tables

The *hierarchy* tables show the disaggregation of data in YSTAFDB, in terms of subsystems and processes, and using UMIS nomenclature[1]. Derivation of process labels from reference material ids, aggregate subsystem module abbreviations, subsystem codes, and process codes is shown in

these *hierarchy* tables, following the procedure outlined in the main text (see the section titled ‘Materials cycles’).

References in this supplementary information document

1. Myers, R. J., Fishman, T., Reck, B. K. & Graedel, T. E. Unified Materials Information System (UMIS): An Integrated Materials Stocks and Flows Data Structure. J. Ind. Ecol., doi: 10.1111/jiec.12730 (2018).
